# Epigenetic Therapeutics Targeting NRF2/KEAP1 Signaling in Cancer Oxidative Stress

**DOI:** 10.3389/fphar.2022.924817

**Published:** 2022-06-09

**Authors:** Shunhao Zhang, Sining Duan, Zhuojun Xie, Wanlin Bao, Bo Xu, Wenbin Yang, Lingyun Zhou

**Affiliations:** ^1^ State Key Laboratory of Oral Diseases, National Clinical Research Center for Oral Diseases, West China Hospital of Stomatology, Sichuan University, Chengdu, China; ^2^ Department of Stomatology, Panzhihua Central Hospital, Panzhihua, China; ^3^ State Key Laboratory of Oral Diseases, National Clinical Research Center for Oral Diseases, Department of Oral and Maxillofacial Surgery, Department of Medical Affairs, West China Hospital of Stomatology, Sichuan University, Chengdu, China; ^4^ Center of Infectious Diseases, West China Hospital of Sichuan University, Chengdu, China

**Keywords:** NRF2, KEAP1, oxidative stress, DNA methylation, histone acetylation, ncRNA, SFN

## Abstract

The transcription factor nuclear factor erythroid 2-related factor 2 (NRF2) and its negative regulator kelch-like ECH-associated protein 1 (KEAP1) regulate various genes involved in redox homeostasis, which protects cells from stress conditions such as reactive oxygen species and therefore exerts beneficial effects on suppression of carcinogenesis. In addition to their pivotal role in cellular physiology, accumulating innovative studies indicated that NRF2/KEAP1-governed pathways may conversely be oncogenic and cause therapy resistance, which was profoundly modulated by epigenetic mechanism. Therefore, targeting epigenetic regulation in NRF2/KEAP1 signaling is a potential strategy for cancer treatment. In this paper, the current knowledge on the role of NRF2/KEAP1 signaling in cancer oxidative stress is presented, with a focus on how epigenetic modifications might influence cancer initiation and progression. Furthermore, the prospect that epigenetic changes may be used as therapeutic targets for tumor treatment is also investigated.

## 1 Introduction

Living organisms are constantly exposed to oxidative stress which is considered as one of the most ubiquitous and significant causes of tumors, and thus have developed an adaptive defense machinery against reactive oxygen species (ROS) to maintain the redox homeostasis at the cellular levels (Suzuki and Yamamoto, 2015). In general, ROS is a collective term that refers to a heterogeneous group of oxygen containing chemically reactive radicals including superoxide (O^2-^), hydroxyl (OH^−^) and hydrogen peroxide (H_2_O_2_), which are unavoidable during cellular aerobic metabolism (D'Autréaux and Toledano, 2007). Contingent upon concentration, ROS are known to exert both beneficial and deleterious effects in cells. At low levels, ROS act as secondary messengers, which are essential for cell survival and various important signal transduction events including nuclear factor-κB (NF-κB) pathway, mitogen-activated protein kinase (MAPK) pathway and activation of p53 (Storz, 2005). On the contrary, elevated generation of intrinsic ROS and/or excessive exposure to extrinsic sources of ROS (for instance, arsenics, nitroaromatics, quinones, X-rays and UV rays) can induce oxidative stress and lead to macromolecules damage, which may be associated with tumorigenesis and cancer progression (Castaldo et al., 2016; He and Jiang, 2016; Kumari et al., 2018). However, the roles of ROS in cancer remains contradictory, varying from distinct cancer types and stages (Schumacker, 2006). Central to the regulation of redox balance is the transcription factor nuclear factor erythroid 2-related factor 2 (NRF2) and its negative regulator kelch-like ECH-associated protein 1 (KEAP1). Under oxidative stress, the interaction between NRF2 and KEAP1 promotes the expression of several antioxidant genes. Thus, activating NRF2/KEAP1 signaling to protect cells from ROS has been widely acknowledged as a promising therapeutic strategy to alter redox equilibration of ROS-related chronic diseases, including cancer (Jeong et al., 2006). Though the roles of NRF2/KEAP1 signaling in other oxidative damage-related diseases including cardiovascular disease, diabetes and neurodegenerative diseases are purely protective, accumulating innovative studies point to a pivotal role for activated NRF2/KEAP1 signaling in promoting cancer progression, metastasis, and resistance to chemo- and radiotherapy, which is essentially arose from the dual nature of ROS in tumor biology (Padmanabhan et al., 2006; Sporn and Liby, 2012; Wang et al., 2016a; Tao et al., 2018). Therefore, activation of NRF2/KEAP1 signaling may lead to distinct even opposite outcomes in cancer and therapeutic strategies targeting this pathway must be cautiously assessed according to the context.

Aberrant NRF2/KEAP1 signaling is correlated with cancer initiation and progression. However, somatic mutations of NRF2 and KEAP1 only occurred in small portion of tumor samples and varied from different clinicopathologic characteristics (Singh et al., 2006; Solis et al., 2010). Thus, alternative mechanisms, other than regulation at DNA level, must govern the NRF2/KEAP1 signaling pathway. Apart from genetic dysregulation, disruption of epigenetic modifications in various signaling pathways can lead to carcinogenesis as well (Zhang et al., 2021; Zhang et al., 2022). Epigenetic changes, namely DNA methylation, histone modifications (methylation, acetylation and phosphorylation) and non-coding RNA (ncRNA) regulation, are covalent and reversible modifications to DNA, histones or mRNA without altering the DNA sequence (Campbell and Tummino, 2014). Intriguingly, it has been reported that epigenetic mechanisms profoundly influence oxidative stress responses through NRF2/KEAP1 signaling and further play an essential role in cancer. For instance, KEAP1 with abnormal promoter methylation contributes to breast tumorigenesis (Barbano et al., 2013a). In addition, histone modifications and ncRNA regulation also occur in NRF2/KEAP1 signaling, resulting in altered expression of target genes (Guo et al., 2015a). In brief, intricate interactions exist between epigenetic alterations and NRF2/KEAP1 signaling, thus selective epigenetic therapeutics targeting NRF2/KEAP1 signaling based on a deeper understanding of contextual and temporal control of NRF2-mediated effects will benefit the development of novel tumor treatments.

Here, we present current advances in respect to the role of NRF2/KEAP1 signaling in cancer oxidative stress, with a particular emphasis on how this pathway can be regulated by epigenetic mechanisms to affect cancer initiation and progression. In addition, the possibility that epigenetic alterations may be potential therapeutic targets for tumor therapy is explored as well.

## 2 NRF2/KEAP1 Signaling and Its Role in Cancer Oxidative Stress

### 2.1 The Molecular Structure of NRF2/KEAP1

**FIGURE 1:**
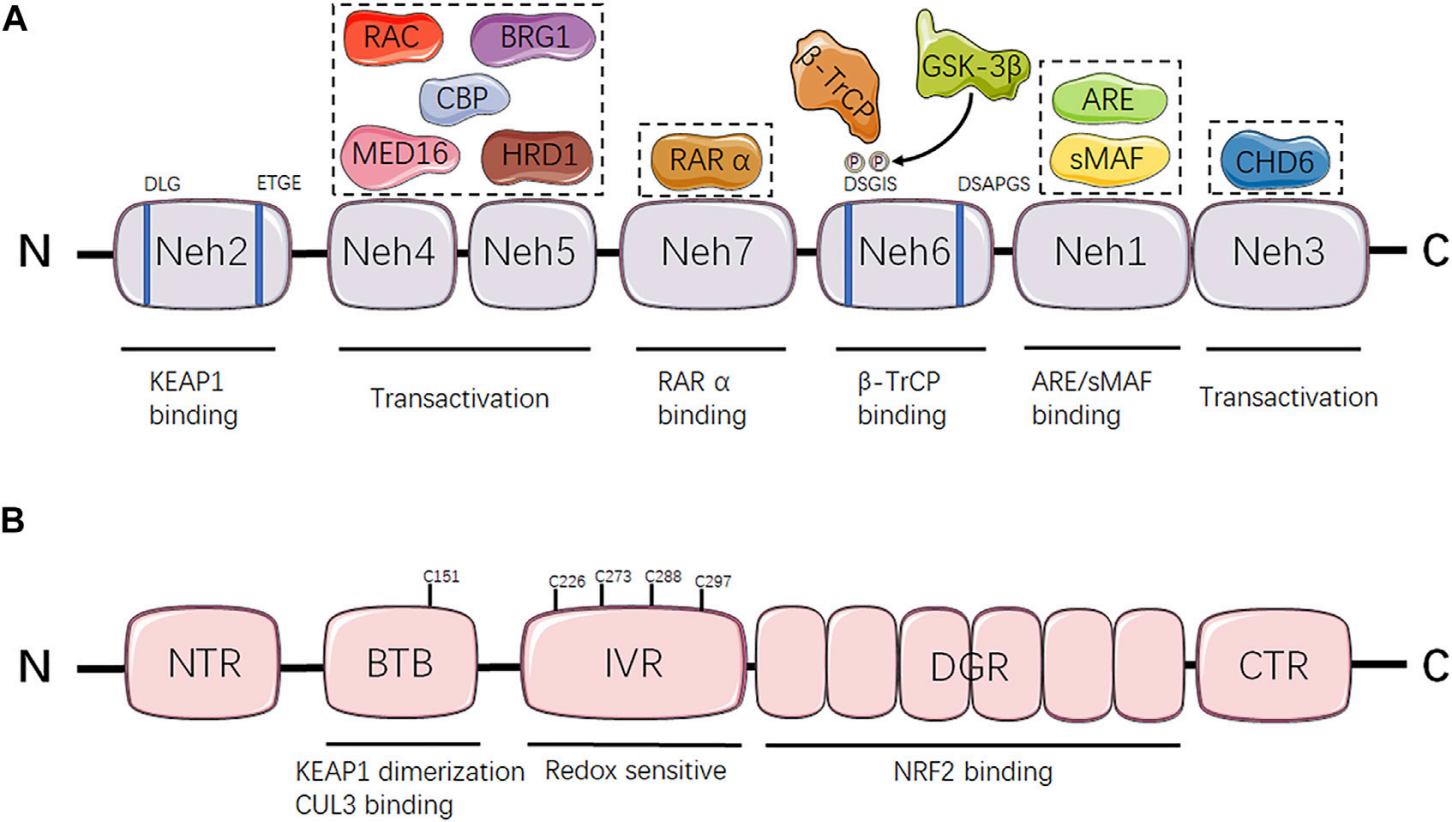
Domain structure and function relationship of NRF2 and KEAP1. **(A)** Schematic representation of NRF2 structure. NRF2-interacting molecules are shown in dash line boxes and placed above their interacting domains. **(B)** Schematic representation of KEAP1 structure. Neh, NRF2-ECH homology; KEAP1, kelch-like ECH-associated protein 1; MED16, mediator complex subunit 16; HRD1: HMG-CoA reductase degradation 1 homolog; RAC, receptor-associated coactivator; BRG1, brahma-related gene 1; CBP, CREB binding protein; RAR α, retinoic acid receptor X receptor α; GSK-3β, glycogen synthase kinase 3-beta; β-TrCP, beta-transducin repeat-containing protein; sMAF, small muscle aponeurosis fibromatosis; ARE, antioxidant response elements; CHD6, chromo-ATPase/helicase DNA binding protein 6; NTR, N-terminus region, BTB, Broad complex/Tramtrack/Bric-a-brac; CUL3, Cullin 3; IVR, intervening region; DGR, double glycine repeat; NRF2, nuclear factor erythroid 2-related factor 2; CTR, C-terminus region.

NRF2 is the genetic product of the NFE2L2 gene, which is located on frequent copy number-gained region of chromosome 2q31.2 and can be genetically altered by copy number amplifications (CNA), promoter demethylation, somatic mutations in ETGE or DLG motifs required for KEAP1 combination, or oncogene-regulated transcription of NRF2 including KRAS^G12D^, BRAF^V600E^ and cMYC^ERT2^, reflecting the universal instabilities of genome inherent to distinct tumors (Cancer Genome Atlas Research, 2012; Imielinski et al., 2012; Berger et al., 2017; Campbell et al., 2016). At the protein level, human NRF2 transcription factor contains 605 amino acids and is composed by seven conserved NRF2-ECH homology (Neh) domains, namely Neh 1–7 with various functions (Higgins and Hayes, 2011) (Figure 1A). Neh 1 contains the Cap-n-Collar (CNC) and basic leucine-zipper (bZIP) domains allowing the binding of NRF2 to small muscle aponeurosis fibromatosis (sMAF) proteins and antioxidant response elements (ARE) (Itoh et al., 1995; McMahon et al., 2006). Neh 2 is the prime regulatory domain located at the N-terminus region (NTR) of NRF2, mediating the KEAP1-dependent degradation of NRF2 through the ETGE and DLG motifs (Itoh et al., 1999). Neh 3–5 are transactivation domains. In greater detail, Neh 3 is located at the C-terminus region (CTR) responsible for promoting NRF2 transcription through interaction with chromo-ATPase/helicase DNA binding protein 6 (CHD6) (Nioi et al., 2005). Both Neh 4 and Neh 5 are essential for NRF2 binding to other transcriptional coactivators, such as mediator complex subunit 16 (MED16), HMG-CoA reductase degradation one homolog (HRD1), receptor-associated coactivator (RAC), brahma-related gene 1 (BRG1) and CREB binding protein (CBP) (Katoh et al., 2001; Zhang et al., 2007; Kim et al., 2013; Wu et al., 2014; Sekine et al., 2016). Neh 6 is a serine-rich domain containing DSAPGS and DSGIS motifs which regulate NRF2 stability. Serine phosphorylation mediated by glycogen synthase kinase 3-beta (GSK-3β) within the DSGIS motif promotes beta-transducin repeat-containing protein (β-TrCP) recognition which leads to NRF2 degradation (Rada et al., 2011; Chowdhry et al., 2013). Neh 7 domain is demonstrated to negatively regulate NRF2 target genes expression through interaction with the retinoic acid receptor X receptor α (RAR α) (Wang et al., 2013).

KEAP1 is a negative regulator of NRF2, acting as an adaptor protein of the Cullin 3 (CUL3) ubiquitin ligase to promote the ubiquitination and subsequent degradation of NRF2 in the proteasomes (Komatsu et al., 2010; Cullinan et al., 2004; Furukawa and Xiong, 2005). KEAP1 contains five distinct domains, including an NTR, a Broad complex/Tramtrack/Bric-a-brac (BTB) domain, an intervening region (IVR), a double glycine repeat (DGR) domain and a CTR (Figure 1B). Among them, the BTB domain is critical for its interaction with CUL3 E3 ubiquitin ligase complex and maintaining KEAP1 dimerization (Furukawa and Xiong, 2005; Ogura et al., 2010). The IVR contains a conserved nuclear export signal (NES) that is implicated in the control of KEAP1 cytoplasmic translocation (Velichkova and Hasson, 2005). Besides, the majority of highly reactive cysteine residues are located at IVR as well, including Cys226, Cys273, Cys288 and Cys297, which are susceptible to modification by oxidants, reactive nitrogen species (RNS) and hydrogen sulfide (H_2_S) resulting in the altered affinity of KEAP1 for NRF2 and eventually NRF2 stabilization or degradation (Dinkova-Kostova et al., 2002; Zhang and Hannink, 2003; Velichkova and Hasson, 2005; Moldogazieva et al., 2018). The DGR domain is composed by six Kelch repeats that are essential for interaction between ETGE/DLG motifs of NRF2 and KEAP1 (Hayes and McMahon, 2009; Komatsu et al., 2010).

### 2.2 The Role of NRF2/KEAP1 Signaling in Antioxidant Defense

Oxidative stress is defined as an imbalance between the elevated ROS generation and antioxidant defense mechanisms. At moderately increased levels, ROS may function as a secondary messenger and control various biological events (Murphy et al., 2011), while excessive generation of ROS has been linked to tissue injury and DNA damage related to neoplastic transformation, maintenance of oncogenic phenotype, cancer cell survival and tumor progression, which emphasize the critical role of precisely regulated redox homeostasis (Khandrika et al., 2009; Idelchik et al., 2017). However, aberrant redox balance is observed in cancer cells. Relative excess of ROS is pro-tumorigenic but cytotoxic at the same time, which indicates that tumor cells adapt to optimize ROS-driven proliferation and avoid senescence, apoptosis or ferroptosis as well through increasing their antioxidant status (Redza-Dutordoir and Averill-Bates, 2016; Reczek et al., 2017; Dodson et al., 2019).

Therefore, the involvement of antioxidant response as an atypical context-dependent driver of tumorigenesis is gaining attention. NRF2 is a master regulator of the cellular antioxidant response through inducing the transcription of a wide range of antioxidant genes (Itoh et al., 1997; McMahon et al., 2001; Hayes et al., 2010). Under normal conditions, KEAP1 are kept binding to Neh 2 domain (ETGE and DLG motifs) of NRF2, which drive NRF2 to CUL3 ubiquitin ligase for subsequent proteasomal degradation, resulting in consistent transcriptional inactivation of NRF2 (Itoh et al., 1999; Nguyen et al., 2003; Tebay et al., 2015). As a cysteine-rich protein that is susceptible to be modified by various electrophilic compounds and oxidant, KEAP1 is an excellent sensor for oxidative stress (Dinkova-Kostova et al., 2002; McMahon et al., 2010). After oxidation of the corresponding cysteine thiols, Cys288 of KEAP1 was found to diminish NRF2 activity, whereas Cys151 was showed to facilitate NRF2 activity (Yamamoto et al., 2008; He and Ma, 2010). Similarly, modification of highly reactive cysteine residues (Cys119 and Cys235) of NRF2 hinder KEAP1 recognition and binding (He and Ma, 2009). Cysteine modifications alter the conformation of KEAP1 and/or NRF2, disrupting the relatively low-affinity interaction between DLG motif and KEAP1, while the association between ETGE motif and KEAP1 remains intact (Tong et al., 2006; Tong et al., 2007). Consequently, the switch from two-site to one-site binding under cellular oxidative stress leads to NRF2 escape from ubiquitination and degradation. Subsequently, NRF2 translocates to the nucleus, where it forms a heterodimer with sMAF protein and binds to ARE *cis*-regulatory sequences to trigger the transcription of target genes (Zhang, 2006). NRF2 downstream genes are mainly involved in intracellular redox-balancing, including sulfiredoxin 1 (SRXN1), peroxiredoxin 1 (PRDX1), thioredoxin reductase (TXNR), thioredoxin 1 (TXN1), glutathione peroxidase (GPx) and glutamate cysteine ligases (GCLC, GCLM), which maintain cellular thioredoxin and glutathione (GSH) levels and reduce ROS levels (Suzuki et al., 2013; Hayes and Dinkova-Kostova, 2014). In specific, NRF2 maintains the proper intracellular reduced GSH/oxidized GSH ratio by regulating the expression of GCLC/GCLM to control GSH synthesis and reduction (Higgins et al., 2009). In addition, NRF2 also controls the pentose phosphate shunt to regulate nicotinamide adenine dinucleotide phosphate (NADPH) availability, which is essential for the reduction of oxidized GSH (Lee et al., 2019; Zhang et al., 2019). Beside the regulation of GSH levels, detoxification of oxidized thiols, peroxide radicals and H_2_O_2_ is under the control of NRF2 as well through fine-tuned expression of GPx2, TXN1 and SRXN1 (Thimmulappa et al., 2002). Heme oxygenase 1 (HMOX1) is another NRF2-regulated cytoprotective enzyme, which catalyze the breakdown of catalase and heme molecules, resulting in the reduction of H_2_O_2_ (Alam et al., 1999).

Though NRF2 has traditionally been regarded as a tumor suppressor due to its cytoprotective role in oxidative stress, increasing evidence demonstrate that NRF2 activation in cancer creates an environment which favors the survival not only of normal cells but also of tumor cells. Several research have revealed that NRF2-related pathways are involved in chemotherapeutic drugs detoxification. Multiple drug metabolic enzymes, including UDP glucuronosyltransferase, aldehyde dehydrogenase and NAD(P)H: quinone reductase (NQO1), are missing in NRF2-deficient mice which results in lower drug detoxification and increased apoptosis, implying that NRF2 is significant for drug metabolism (Aleksunes and Klaassen, 2012; Bai et al., 2016). Importantly, advanced-stage lung cancer cell lines overexpress NQO1. As a transcriptional target of NRF2, upregulated NQO1 can be attenuated by NRF2 blockade to sensitize lung cancer cells to several conventional chemotherapeutic drugs including etoposide, doxorubicin and cisplatin (Wang et al., 2008a). Furthermore, ATP-binding cassette transporters that govern the elimination of chemotherapeutic drugs in cancer cells are regulated by NRF2 as well, conferring the chemoresistance phenotype on tumor (Singh et al., 2010). To conclude, NRF2 is correlated with reduced apoptosis in cancer cells that are exposed to chemotherapeutic agents. In addition to chemoresistance, NRF2 activity is found to be associated with cancer metastasis. As mentioned above, NRF2-regualted cytoprotective enzyme HMOX1 is critical for heme catabolism. Lignitto et al. (2019) discovered that HMOX1 and the cellular heme level modulated the transcription factors BTB and CNC homology 1 (BACH1), which dictated lung cancer metastasis. Besides, NRF2 also can activate a metastatic program through the RhoA/ROCK pathway in breast cancer (Zhang et al., 2016a). In brief, their study demonstrated that NRF2 are involved in promoting cancer metastasis through multiple mechanisms. Therefore, the boundaries between NRF2 negative and positive effects should be cautiously defined in terms of cancer types and stages (Menegon et al., 2016).

## 3 Epigenetic Regulation of NRF2/KEAP1 Signaling in Cancer

DNA can substantially encode all the biological information an organism needed. Apart from this structure, epigenetic regulation of gene expression also plays a critical role in cell differentiation and mammalian development (Iacobuzio-Donahue, 2009; Wainwright and Scaffidi, 2017). The mechanism of epigenetic modification is the molecular biological process that affects cell behavior through alterations in gene expression without modifying DNA sequences (Yen et al., 2016). Recent research about the correlation between carcinogenesis and epigenetic change suggested that cancer can be induced by abnormal epigenetic alterations in genome sequences at multiple stages (Guo et al., 2015b).

A great deal of previous research into the NRF2/KEAP1 signaling focuses on the dual roles of NRF2 in cancer (Menegon et al., 2016; Sun et al., 2017; Schmidlin et al., 2021). As mentioned above, NRF2/KEAP1 signaling commonly acts as cellular defensive machinery under oxidative stress, which is a vital factor correlated with neoplastic diseases (Sporn and Liby, 2012; Rojo de la Vega et al., 2018). Epigenetic modification has been a crucial mechanism for regulating the NRF2/KEAP1 signaling pathway under oxidative stress, including DNA methylation, histone acetylation and ncRNAs (Bhat et al., 2018; Wei et al., 2019). The following paragraph aims to outline the mechanism of NRF2/KEAP1 epigenetic modifications in cancer (Table 1).

**TABLE 1:** Epigenetic modification and potential targets of NRF2/KEAP1 signaling.

Epigenetic mechanisms	Cancer type or model	Dietary agent	Molecular mechanism	Downstream effects	Reference
DNA methylation	Prostate cancer	sulforaphane (SFN)	DNMTs↓	Formation of DNA adducts↓	(Zhang et al., 2013; Su et al., 2014; Li et al., 2018b; Soundararajan and Kim, 2018)
			DNMT1, 3a,3b↓	Cell transformation and development↓	
		3,3’-diindolylmethane (DIM)	DNMT1, 3a, 3b↓	Cell proliferation and prostate carcinogenesis↓	(Wu et al., 2013)
				Apoptosis↑	
		γ-Tocopherol-rich mixture of tocopherols (γ-TmT)	DNMT1, 3a, 3b↓	Prostate carcinogenesis↓	(Huang et al., 2012)
		Corosolic acid (CRA)	DNMT1, 3a, 3b↓	Prostate carcinogenesis↓	(Yang et al., 2018a)
		Curcumin	DNMT1, 3a, 3b↓	Prostate carcinogenesis↓	(Khor et al., 2011)
				Anti-oxidative stress and cellular defense pathway↑	
		Astaxanthin (AST)	DNMT1, 3a, 3b↓	Cell viability↓	(Yang et al., 2017)
				Cellular transformation↓	
	Mouse skin JB6 P+ cell	Apingenin	DNMT1, 3a, 3b↓	Anticancer effects↑	(Pandey et al., 2012; Tseng et al., 2017)
		Delphinidin	DNMT1, 3a↓	Cell cycle arrest, differentiation, and cell death↑	(Kuo et al., 2019)
		Pelargonidin	DNMT1, 3b↓	Neoplastic transformation↓	(Li et al., 2019)
		Reserpine	DNMT1, 3a, 3b↓	Neoplastic transformation↓	(Hong et al., 2016)
				Cellular protection↑	
		Tanshinone IIA (TIIA)	DNMT1, 3a, 3b↓	Cellular transformation↓	(Wang et al., 2014; Yang et al., 2020b)
				Anticancer effects↑	
		Taxifolin (TAX)	DNMT1, 3a, 3b↓	Anticancer effects↑	(Kuang et al., 2017)
		Ursolic acid (UA)	DNMT1, 3a↓	Anticancer effects↑	(Kim et al., 2016; Wang et al., 2018a)
		Astaxanthin (AST)	DNMT1, 3a, 3b↓	Cellular transformation↓	(Yang et al., 2018b)
				Anticancer effects↑	
		Fucoxanthin	DNMTs ↓	Cellular transformation↓	(Yang et al., 2018b)
				ARE-luciferase activity↑	
	Colon cancer	Luteolin(LUT)	DNMT1, 3a, 3b↓	Apoptosis and cytotoxicity↑	(Zuo et al., 2018; Kang et al., 2019)
	Breast cancer	Resveratrol	DNMT3b↓	E2-induced breast carcinogenesis↓	(Singh et al., 2014)
	Non-small cell lung cancer	5-aza-2'-deoxycytidine	DNMTs ↓	Patients survival↑	(Wang et al., 2008b; Chien et al., 2015; Guo et al., 2015b; Gao et al., 2019)
				Lymph node metastasis↓	
	Hepatoellular carcinoma	Fucoxanthin	DNMTs ↓	Cellular transformation↓	(Yang et al., 2018b)
				ARE-luciferase activity↑	
		Fumonisin B	DNMTs ↓	ROS production ↑	(Arumugam et al., 2021)
				Cell membrane damage↑	
Histone acetylation	Prostate cancer	sulforaphane (SFN)	HDAC1, 4, 5, 7↓	Cell transformation and development↓	(Zhang et al., 2013)
		Corosolic acid (CRA)	HDAC1, 2, 3, 4, 7, 8↓	Prostate carcinogenesis↓	(Chen et al., 2012; Yang et al., 2018a)
		3,3’-diindolylmethane (DIM)	HDACs↓	Detoxification and excretion of chemicals↑	(Lewinska et al., 2017)
				Cell Proliferation and prostate carcinogenesis↓	
	Mouse skin JB6 P+ cell	sulforaphane (SFN)	HDAC1, 2, 3, 4↓	Neoplastic transformation↓	(Su et al., 2014)
		Delphinidin	HDACs↓	Cell transformation↓	(Kuo et al., 2019)
				Cellular protection↑	
		Pelargonidin	HDAC1, 2, 3, 4, 7↓	Cell transformation↓	(Li et al., 2019)
				Cellular protection↑	
		Taxifolin (TAX)	HDAC1 to 8 ↓	Anticancer effects↑	(Kuang et al., 2017)
		Ursolic acid (UA)	almost all HDACs↓	Anticancer effects↑	(Kim et al., 2016)
		Corosolic acid (CRA)	HDACs↓	Cell cycle arrest, autophagy and apoptosis↑	(Jin et al., 2021)
	Non-small cell lung cancer	Luteolin (LUT)	HDAC1, 2, 3, 6, 7↓	Cell viability and growth capacity↓	(Zuo et al., 2018)
	Colorectal cancer	sulforaphane (SFN)	Transcriptional regulator complex↓	Cellular antioxidant and detoxification↓	(Sharma et al., 2005; Saxena and Sharma, 2010; Meeran et al., 2012)
	Breast cancer	Glucocorticoids (GC)	histone acetylation at ARE and decrease NRF2 transcriptional activation↓	NRF2-mediated antioxidant response↓	(Alam et al., 2017)
Non-coding RNA	Hepatocellular carcinoma	phenethyl-isothiocyanate (PEITC)	miR-200c↓	ROS production↓	(Xiao et al., 2012; Gerhauser, 2013)
			miR-141↓	Apoptotic cell death↑	
	Prostate cancer		miR-200a-3p/141-3p	Tumor metastases and tumor burden↓	(Mateescu et al., 2011; Lan, 2012; Jiang et al., 2013; Cortez et al., 2014)
				Reactive oxygen overproduction↓	
				Cellular protection↑	
	Breast cancer	Resveratrol	miR-93↓	E2-induced breast carcinogenesis↓	(Singh et al., 2014)
		Victim C	miR-93↓	Apoptosis, cellular protection and colony formation↑	(Mense et al., 2009; Singh et al., 2012; Singh et al., 2013; Wang et al., 2016a)
			miR-153↓		
	Hepatocellular carcinoma	Apigenin	miR-101↓	Apoptosis and chemo-sensitization↑	(Liu et al., 2016; Gao et al., 2017)
		Polydatin	miR-200a↓	Antioxidant and antiinflammation↑	(Zhao et al., 2018)
				Lipid deposition↓	

### 3.1 DNA Methylation

**FIGURE 2:**
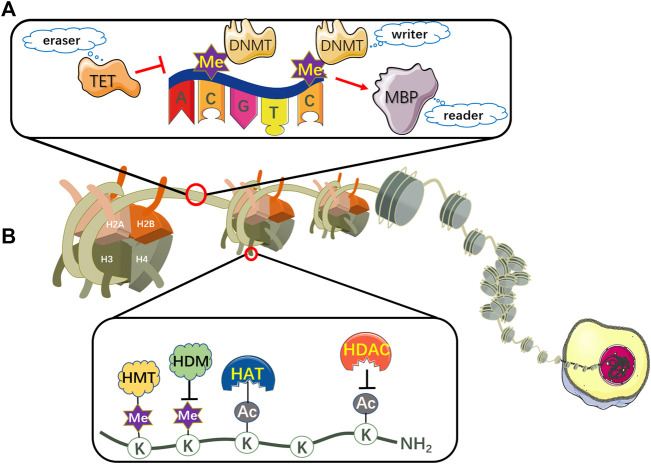
The mechanism of DNA methylation and histone modifications. **(A)** DNA can be epigenetically modified by DNMT-based methylation. DNMTs are involved in catalyzing a methyl group to CpG dinucleotides, thus representing writers in epigenetic modifications. MBPs serve as readers to recognize and bind to the methylated CpG sites. TET proteins function as erasers to remove the epigenetic label of 5mC. **(B)** Nucleosomes comprise eight histone proteins including two copies of H2A, H2B, H3 and H4. Histone acetylation is maintained by the coordination of HATs and HDACs, which acetylate or deacetylate the lysine residues respectively in the N-terminal tails of histones protruding from the octamer. Histone lysine methylation is regulated by HMTs and HDMs, which transfer or remove highly processive methyl addition to the lysine. TET, ten-eleven translocation; DNMT, DNA methyltransferase; MBP, methyl-CpG binding protein; Me, methyl group; HAT, histone acetyltransferase; HDAC, histone deacetylase; HMT, histone methyltransferase; HDM, histone demethylase; K, lysine residue; AC, acetyl group.

DNA methylation, the most representative chemical modification in the epigenome, usually occurs in a cytosine-guanine dinucleotide (CpG) site through covalent addition of a methyl group at the 5-carbon position of the cytosine base to form 5-methylcytosine (5mC) (Skvortsova et al., 2019; Wang et al., 2021). These CpG sites are not distributed randomly across the human genome, conversely, concentrated in so-called CpG islands situated in gene promoter regions or non-transcribed regions with large repetitive sequences (Erdmann et al., 2016). CpG islands methylation can turn off tumor suppressor genes, which is related to deregulation of the transcriptome and cellular pathways (Choi et al., 2017). Additionally, the overall DNA methylation modification can be iconically compared to three processes: writing, reading and erasing (Wang et al., 2021) (Figure 2A).

#### 3.1.1 The Writing Process in DNA Methylation Pattern

DNA methylation is routinely mediated by DNA methyltransferases (DNMTs), a class of enzymes involved in delivering a methyl group from S-adenosyl methionine (SAM) to the 5-carbon position of cytosine ring to propel the DNA methylation process, thus representing “writers” in epigenetic modifications (Toh et al., 2017). The target cytosine gets off the DNA double-helical structure and binds to the active site of DNMT. Then the thiolate in the cysteine residue of DNMT functions as a strong nucleophile, attacking the 6-carbon atom in the pyrimidine ring of cytosine to construct a covalent bond between the thiolate atom and the 6-carbon atom. Subsequently, the sulfonium methyl of the cofactor SAM transfers to the 5-carbon of the cytosine ring. Afterwards, β-elimination takes place between the 5-carbon and 6-carbon bond to dislodge DNMTs from the methylated cytosine (Erdmann et al., 2016; Poh et al., 2016).

To date, four active enzymes have been identified as significant members of the DNMT family: DNMT1, DNMT3a, DNMT3b and DNMT3L. Among all, DNMT1 is involved in the maintenance of the global DNA methylation pattern by duplicating DNA methylation to newly biosynthesized DNA, predominantly targeting hemimethylated DNA during S phase (Medina-Franco et al., 2015; Köhler and Rodríguez-Paredes, 2020). DNMT3a and DNMT3b function as *de novo* DNA methyltransferases to catalyze the methylation of unmethylated genomic regions with the help of DNMT3L (Schmitz et al., 2019). In mammals, DNMT3a and DNMT3b initially establish the methylation pattern during embryonic development (Zocher et al., 2021). Specifically, DNMT3L can activate the enzymatic activity of DNMT3a/DNMT3b and gradually enlarge the methylation pattern on DNA sequences (Agrawal et al., 2018). Furthermore, many chemopreventive chemicals targeting DNMT have been found and will be discussed in section 4.1.

#### 3.1.2 The Reading Process in DNA Methylation Pattern

The methyl-CpG binding proteins (MBPs), which serve as “readers” to recognize and bind to the methylated CpGs sites, subsequently coordinating the crosstalk among DNA methylation, histone acetylation and chromatin remodeling to activate downstream regulatory elements (Zafon et al., 2019). So far, three MBP families have been identified, including the methyl binding domain (MBD) family, the zinc finger/Kaiso family, and the SET and RING associated (SRA) domain family (Bartels et al., 2011; Jobe and Zhao, 2017).

#### 3.1.3 The Erasing Process in DNA Methylation Pattern

The ten-eleven translocation (TET) family proteins are regarded as demethylases which include three family members: TET1, TET2 and TET3. The TET proteins sequentially oxidize 5mC into 5-hydroxymethylcytosine (5hmC), 5-formylcytosine (5fC) and 5-carboxylcytosine (5caC), then ultimately reverse it to an unmethylated cytosine residue. Therefore, they function as “erasers” to remove the epigenetic label of 5mC (Cheng et al., 2016; Morgan et al., 2018). TET-mediated demethylation is triggered by passive and active mechanisms, namely the DNA replication-dependent passive pathway and the thymine DNA glycosylase (TDG)-initiated base excision repair (BER) active pathway (Wu and Zhang, 2017).

When DNMT1 fails to methylate the newly synthesized DNA sequence, the deposition of 5hmC may activate a passive mechanism by inhibiting the enzymatic activity of DNMT1 and thus impede DNMT1-mediated methylation process (Ji et al., 2014). In addition, the active DNA demethylation refers to an enzymatic process in which 5mC and other derivatives are oxidized through TDG-initiated BER pathway by TET proteins in the local genome. Specifically, TDG is a DNA mismatch repair enzyme that recognizes and excises the oxidized cytosine base of 5fC and 5caC, which leaves an abasic site for BER and eventually results in DNA demethylation (Rasmussen and Helin, 2016). Compared to the passive pathway, the active pathway is more rapid and may cause DNA damage and genomic instability (Wu and Zhang, 2017).

### 3.2 Histone Modifications

In addition to DNA methylation, histone modifications including acetylation and methylation can regulate gene expression by influencing chromatin structure. The nucleosome, the basic subunit of chromatin, encompasses eight histone proteins with two copies each of H2A, H2B, H3 and H4, whose lysine residues in the N-terminal tails of histones protruding from the octamer contain sites for post-translational modifications (PTMs). They can either be acetylated or methylated by histone acetyltransferases (HATs) or histone methyltransferases (HMTs), as well as deacetylated or demethylated by histone deacetylases (HDACs) or histone demethylases (HDMs) (Li et al., 2016; Shah, 2019) (Figure 2B). Here, we review two pivotal histone modifications in NRF2/KEAP1 signaling pathway.

#### 3.2.1 Histone Acetylation

There is compelling evidence that acetylation process plays an essential role in the epigenetic regulation of chromatin structure and gene transcription. HAT acetylates histones by adding acetyl groups to lysine residues in the lysine-rich N-terminal tails, which causes nucleosome relaxation and gives DNA access to the transcriptional protein complex (Meeran et al., 2010). Conversely, the deacetylation is catalyzed by HDACs and the overexpression of HDACs is closely correlated with transcriptional repression of tumor suppressor genes, leading to dysregulation of cell cycle, proliferation, differentiation and apoptosis in malignancies (Kaufman-Szymczyk et al., 2015; Su et al., 2018a; Luan et al., 2019). To date, 18 mammalian HDACs have been identified and preliminarily classified into four classes based on their sequence homologies and catalytic mechanism (Yoon and Eom, 2016; Yoon et al., 2019): 1) class I (HDAC1-3 and 8); 2) class II (HDAC4-7, 9 and 10); 3) class III (Sirt1-7); 4) class IV (HDAC11). Class II HDAC is further divided into two subgroups including class IIa (HDAC4, 5, 7 and 9) and class IIb (HDAC6 and 10) (Hull et al., 2016). HDACs are regarded to be overexpressed in cancer cells, which is correlated with poor clinical outcomes in various cancers, such as gastric (Calcagno et al., 2019), colon (Kim et al., 2019) and breast cancer (Guo et al., 2018). In cell-free biochemical assay, HDAC1-3 and 6 are more sensitive to enzyme substrates with peptides containing simple acetyl-lysine than other isoforms, thus will be discussed below (Ho et al., 2020).

HDAC1 and HDAC2 are regarded as core HDACs in that they directly impact gene transcription and exist in virtually all species (Kelly and Cowley, 2013). In addition to histone proteins, they also deacetylate crucial non-histone proteins involved in transcriptional regulation (Adler and Schmauss, 2016; Liu et al., 2019). For instance, excessive deacetylation of tumor suppressor protein p53 suppresses its influences on cellular activities such as cell cycle arrest, apoptosis and even autophagic regulatory (Mrakovcic et al., 2019). Similarly, HDAC3 is also omnipresently expressed and involved in histone deacetylation. The difference lies in the C-terminal tails, where HDAC3 binds to the nuclear NCoR/SMRT complexes and therefore acquires the catalytic function (Emmett and Lazar, 2019). A variety of studies have shown that class I HDACs inhibit ARE-dependent gene expression. Specifically, NF-κB subunit p65 antagonizes NRF2-ARE pathway *via* depriving NRF2 of CREB binding protein (CBP), a member of HAT, and recruiting HDAC3 to the ARE element, hence mediating the necrotic cell death in response to oxidative stress (Guo et al., 2020). On the other hand, HDAC3 also regulates KEAP1/NRF2 in tumor cells through modulating the expression of miR-200a as well (Zhao et al., 2019).

Unlike other zinc-dependent type II HDACs, HDAC6 is primarily localized in the cytoplasm, thus its biological functions are more related to the acetylation of non-histone proteins such as α-tubulin, HSP90 and cortactin with two functional catalytic domains (DD1 and DD2) and a C-terminal ubiquitin-binding zinc finger domain (ZnF-UBP domain), which regulates ubiquitination-mediated degradation (Ho et al., 2020; Liu et al., 2021). According to recent research, DD1 and DD2 are responsible for the deacetylation of substrates bearing acetyl-lysine at C-terminus residues or peptides with internal acetyl-lysine residues, respectively (Li et al., 2018a; Kutil et al., 2019). Marc Kästle et al. (Kästle et al., 2012) reported that HDAC6 participates in preventing cellular damage through proteasome inhibition. During the accumulation of ubiquitinated proteins, HDAC6 deacetylates p38 properly and facilitates the subsequent phosphorylation of p38, leading to the activation of NRF2 and the induction of anti-inflammatory protein HO-1 transcription.

#### 3.2.2 Histone Methylation

The methylation or demethylation of histones occurs by adding or removing various methyl groups on the basic amino acids lysine and arginine. Similar to DNA methylation, histone methylation also uses SAM as the methyl group donor with the help of HMT (Kim et al., 2017). Depending on the residue methylated loci and degree, histone methylation leads to either gene activation or repression (Hyun et al., 2017; Gong and Miller, 2019). For example, the lysine methyltransferase EZH2 catalyzes the trimethylation of histone H3 lysine 27 (H3K27me3), and lysine methyltransferase 7 (SetD7) targets histone H3 lysine 4 (H3K4). It is reported that EZH2 downregulation in lung cancer leads to the reduction of H3K27me3 at NRF2 promoter area and increases NRF2 transcription eventually (Li et al., 2014), whereas SetD7 can activate the antioxidant NRF2/KEAP1 pathway by elevating H3K4 methylation in prostate cancer cells (Wang et al., 2018a).

### 3.3 Regulation of ncRNAs

**FIGURE 3:**
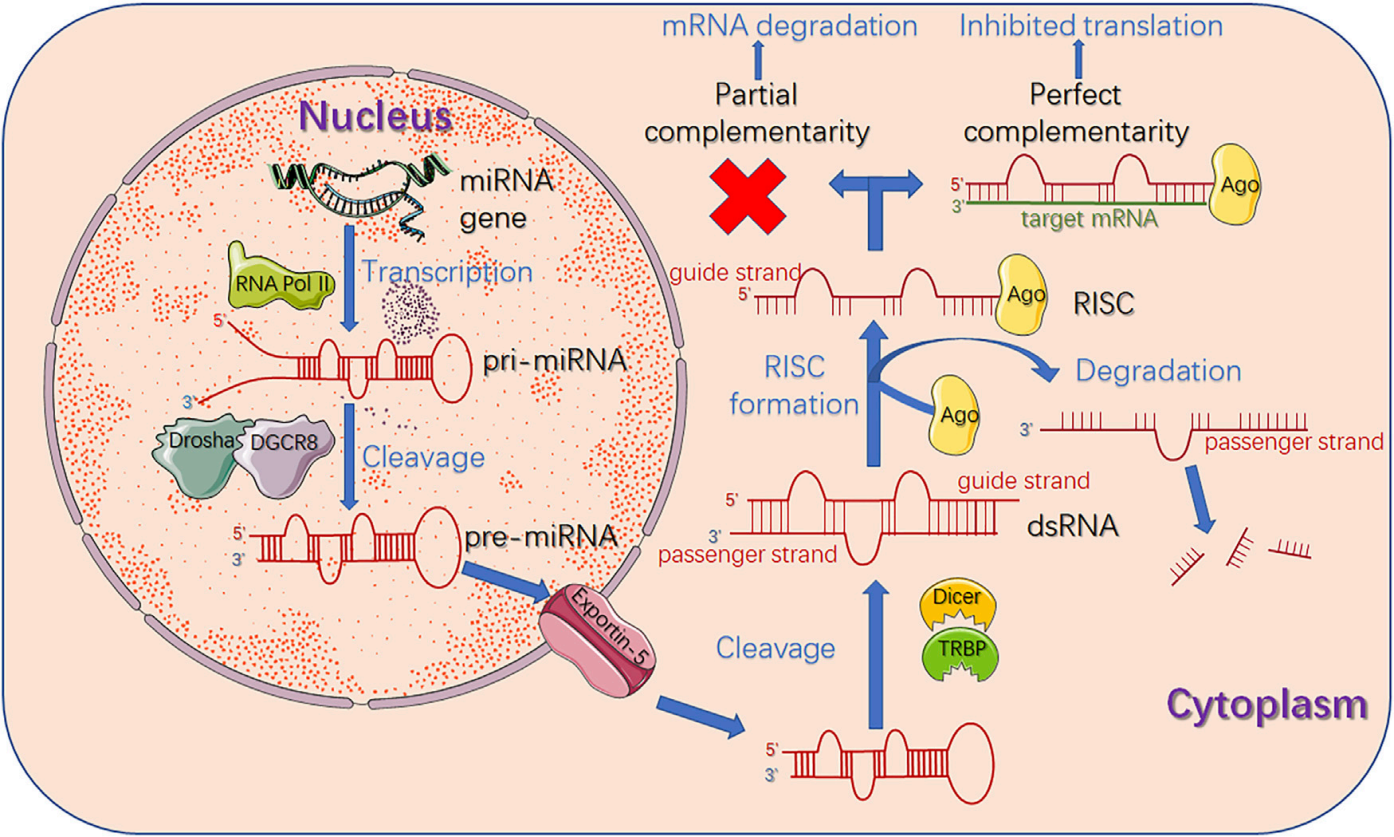
Biogenesis and functions of miRNA. At the beginning, miRNA gene is transcribed to pri-miRNA by RNA polymerase II. Then RNAse III Drosha and its cofactor protein DGCR8 bind to pri-miRNA to generate pre-miRNA through enzymatic cleavage. Subsequently, pre-miRNA is exported to cytoplasm *via* Exportin five and finally cleaved into dsRNA by Dicer/TRBP complex, which can be separated into guide strand and passenger strand. After the cleavage of passenger strand, the remanent guide strand binds to Ago to form RISC, which mediates the recognition of target mRNA. Judging by the degree of complementarity between the sequences of miRNA and the target mRNA, this leads to either inhibited translation or mRNA degradation. miRNA: microRNA; RNA Pol II: RNA polymerase II; pri-miRNA: primary miRNA; pre-miRNA: precursor miRNA; dsRNA: double-stranded RNA; Ago: argonaute protein; RISC: RNA-induced silencing complex.

The ncRNA network is established by microRNAs (miRNAs), long noncoding RNAs (lncRNAs) and circular RNAs (circRNAs), modulating a myriad of cellular mechanisms related to cancer initiation and progression at transcriptional and post-transcriptional levels (Fabrizio et al., 2018; Yu et al., 2021). Particularly, tremendous progress has been made in various tumor treatments using nanoparticle-conjugated miRNA mimetics (Rupaimoole and Slack, 2017). Therefore, we will focus on the regulation mechanism of miRNA in the following section. MiRNA, an important class of short ncRNA molecules that regulates gene expression at the post-transcriptional level by binding to the 3′-untranslated region (3′-UTR) of specific mRNA, negatively regulates the KEAP1/NRF2 pathway through inhibiting specific mRNA translation or inducing mRNA degradation by sequence complementarity (Fabrizio et al., 2018). Most miRNA synthesis is carried out through the canonical pathway (Figure 3). In this respect, miRNA genes are initially transcribed to primary miRNAs (pri-miRNAs) aided by RNA polymerase II (Amirkhah et al., 2019). Pri-miRNAs contain some stem-loop structures with a poly-A tail at the 3′end and a cap at the 5′end. It is then converted to precursor miRNAs (pre-miRNAs) in the nucleus by an enzymatic complex containing RNAse III Drosha and the double-strand binding protein DGCR8 (Yang et al., 2020a). Subsequently, it is exported to the cytoplasm *via* nuclear receptor Exportin-5 and finally cleaved into small double-stranded RNAs (dsRNAs) by Dicer/TRBP complex, which can be separated into guide strand and passenger strand (Saliminejad et al., 2019; Treiber et al., 2019). After the cleavage of passenger strand, the remanent guide strand then binds to argonaute protein (Ago) and generates the RNA-induced silencing complex (RISC), which specifically binds to the 3′-UTR of the target mRNA through complementary base pairing. Judging by the degree of complementarity between the sequences of the miRNA and the target mRNA, this leads to either inhibited translation or mRNA degradation (Hosseinahli et al., 2018).

The recent scientific evidence shows that several miRNAs affect the NRF2/KEAP1 signaling by directly regulating the NRF2 expression or indirectly modulating KEAP1 and other upstream factors of the pathway (Panieri and Saso, 2019; Ashrafizadeh et al., 2020). For example, miR-200a stimulates the NRF2/KEAP1 signaling by suppressing KEAP1 to decrease ROS concentration in breast cancer cells (Cloer et al., 2019; Bono et al., 2021), while a few identified miRNAs including miR144/153/27a/142-5p directly modulate NRF2 dependent redox homeostasis by suppressing NRF2 gene expression in neuronal cells (Narasimhan et al., 2012). It is also reported that during carcinogenesis, the decreased miR144/153/27a/142-5p as well as miR-200a contribute to the upregulated NRF2 levels and activate phosphorylation activity, which increases cell survival and facilitates tumor growth (Zimta et al., 2019).

## 4 Therapeutic Strategies Targeting Epigenetic Modifications of NRF2/KEAP1 Signaling in Cancer

### 4.1 Targeting DNA Methylation

**FIGURE 4:**
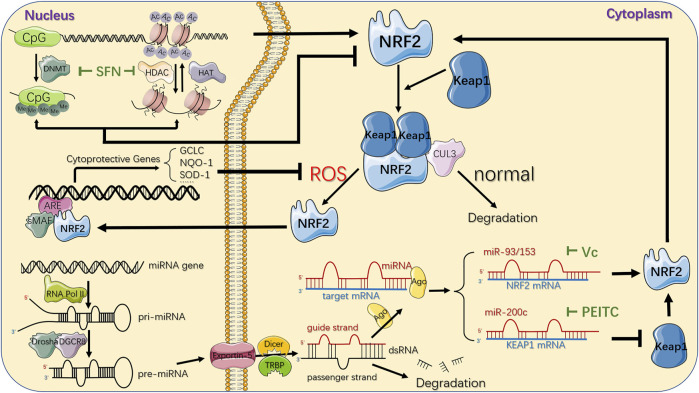
Epigenetic mechanisms and therapeutic strategies targeting NRF2/KEAP1 pathway. CpG sites of NFE2L2 promoter region can be epigenetically modified by DNMT-based methylation, while histone acetylation is maintained by the coordination of HATs and HDACs, which acetylate or deacetylate the lysine residues in the N-terminal tails of histones protruding from the octamer respectively. SFN, a natural phytochemical, primarily attenuates both DNMTs and HDACs, individually suppressing DNA hypermethylation and histones deacetylation, ultimately upregulating NRF2. Another epigenetic modification of NRF2/KEAP1 pathway deregulation in cancer comes from several miRNAs that downregulate or upregulate NRF2 protein expression in the cytoplasm by directly targeting 3′-UTR sequences of NRF2 or KEAP1 mRNA. The important phytochemicals and mechanisms interacting with NRF2/KEAP1 pathway are displayed. For instance, Vitamin C could inhibit miR-93 to upregulate NRF2 mRNA while PEITC could inhibit miR-200c to downregulate KEAP1 mRNA, jointly resulting in activation of NRF2/KEAP1 pathway. Under normal conditions, NRF2 is bound to KEAP1, ubiquitylated by CUL3 ubiquitin ligase, and turns into proteasomal degradation. In response to oxidative stress, KEAP1 is suppressed and leads to NRF2 stabilization. Subsequently, it translocates to the nucleus where it binds to the ARE in the genome with sMAF proteins, and eventually activates the transcription of its downstream genes, which are mainly involved in the redox homeostasis of the cell. DNMT: DNA methyltransferase; Me: methyl group; HAT: histone acetyltransferase; HDAC: histone deacetylase; AC: acetyl group; miRNA: microRNA; RNA Pol II: RNA polymerase II; pri-miRNA: primary miRNA; pre-miRNA: precursor miRNA; Ago: argonaute protein; ARE: antioxidant response element; SFN: Sulforaphane; PEITC: Phenethyl-isothiocyanate.

Recent studies have shown that there are various natural chemical ingredients or phytochemicals in vegetables and medicinal herb exerting anti-carcinogenic effects *via* epigenetic regulation of NRF2, among which the isothiocyanates (ITC), a bioactive present enzymatically hydrolysed from glucosinolates (GLs) in Brassicaceae plant family (Kołodziejski et al., 2019), was considered as one of the most successful, naturally occurring and dietary chemopreventive compound (de Figueiredo et al., 2015). Among the numerous ITC members, sulforaphane (SFN) and phenethyl-isothiocyanate (PEITC) exhibit strong anti-inflammatory and anti-carcinogenic activity (Saw et al., 2011), closely related to the decreased incidence of cancers *via* influencing proliferation, apoptosis and cell cycle (Clarke et al., 2011; de Figueiredo et al., 2015; Johnson et al., 2017; Zuo et al., 2018) (Figure 4).

Masses of research have revealed that SFN upregulated both expression and stabilization of NRF2 primarily through its role as a DNMT inhibitor (Su et al., 2018b), exerting powerful anti-cancer effect in the esophageal cancer (Lu et al., 2021), breast cancer (Myzak et al., 2006) and cervical cancer (Myzak et al., 2006). In particular, in both *in vitro* and *in vivo* prostate cancer models (Zhang et al., 2013; Su et al., 2014; Li et al., 2018b; Soundararajan and Kim, 2018), the significant attenuation of DNMT1 and DNMT3a contributed to a decrease in the methylated CpG ratio in the NFE2L2 promoter region in an SFN dose- and time-dependent manner, thus increasing NRF2 expression and consequently increasing the transcription of its target genes such as NQO1 and catechol-O-methyltransferase (COMT) to inhibit the formation of DNA adducts against reactive oxygen damage, promoting cell apoptosis and cell cycle disorders (Nair et al., 2010; Zhang et al., 2013; Negrette-Guzmán et al., 2017). However, although ITCs can trigger the activation of NRF2 dependent genes, it is believed that excessive activation of NRF2 is associated with tumor progression and increased resistance to chemotherapeutics, indicating the complicated roles of ITC in cancer (Ernst et al., 2011). A clinic trial conducted by OHSU Knight Cancer Institute (NCT01228084) proved that SFN may prevent or slow the growth of recurrent prostate cancer, essentially without severe adverse events.

Additionally, various chemopreventive compounds were reported to serve as DNMT inhibitors, reverse NFE2L2 hypermethylation in parallel mechanism to SFN and consequently regulate NRF2/KEAP1 pathway to exert anticancer effects, such as 3,3′-diindolylmethane (DIM) (Wu et al., 2013), γ-Tocopherol-rich mixture of tocopherols (γ-TmT) (Huang et al., 2012), Corosolic acid (CRA) (Yang et al., 2018a) and Curcumin (Khor et al., 2011) in the prostate cancer, and Apigenin (Pandey et al., 2012; Tseng et al., 2017), Delphinidin (Kuo et al., 2019), Pelargonidin (Li et al., 2019), Reserpine (Hong et al., 2016), Tanshinone IIA (TIIA) (Wang et al., 2014; Yang et al., 2020b), Taxifolin (TAX) (Kuang et al., 2017) and Ursolic acid (Kim et al., 2016; Wang et al., 2018b) in the mouse skin epidermal (JB6 P+) cell. Especially, Astaxanthin (AST), a red dietary carotenoid, could significantly increase the mRNA expression of DNMT3a at a low concentration but decrease the expression and activation of DNMT1, 3a and 3b at a relatively high concentration, while steadily attenuated NQO1 expression *via* NRF2/KEAP1 pathway in dose-dependent manners, ultimately reducing the cell viability and cellular transformation respectively in the prostate cancer cells (Yang et al., 2017) and mouse skin JB6 P+ cells (Yang et al., 2018b). It reminds us of the importance that the phytochemicals’ effect of concentration or duration on its effectiveness. The same mechanism or effectiveness could happen with the treatment of Luteolin (LUT) in colon caner (Zuo et al., 2018; Kang et al., 2019), Resveratrol in breast cancer (Singh et al., 2014), Fucoxanthin in both hepatoma carcinoma and mouse skin epidermal cells model (Yang et al., 2018b).

KEAP1 methylation also has a great impact on cancer biology by regulating NRF2/KEAP1 signaling. Aberrant hypermethylation in KEAP1 promoter region was the most common alteration found in nearly half of the non-small cell lung cancer (NSCLC) cases (Muscarella et al., 2011a), and has been proven to be associated with poor prognosis in various cancers including malignant glioma, breast cancer and pancreatic cancer (Muscarella et al., 2011b; Barbano et al., 2013b; Zhang et al., 2016b). Numbers of research has revealed that 5-aza-2′-deoxycytidine (5-aza) treatment demethylated the CpG sites in the KEAP1 promoter region, synergistically contributing to KEAP1 overexpression, NRF2 degradation and inactivation of various relevant signal pathways (Wang et al., 2008b; Guo et al., 2015c; Gao et al., 2019), which were related to enhanced survival and reduced lymph node metastasis of NSCLC patients (Chien et al., 2015). Contrary to 5-aza, Fumonisin B, a common toxic mycotoxins of cereal grains, activated NRF2/KEAP1 pathway by hypermethylating CpG islands in KEAP1 gene, consequently enhancing ROS production along with promoting cell membrane damage in human hepatoma cells (Arumugam et al., 2021). In conclusion, the expression and activation of KEAP1 could be regarded as an effective therapeutic strategy for advanced human cancers.

### 4.2 Targeting Histone Acetylation

According to previous research, numbers of chemopreventive compounds could affect histone acetylation mainly by regulating the activation or expression of HDACs involved in chromatin remodeling, gene expression and NRF2/KEAP1 signaling (Jabbarzadeh Kaboli et al., 2020), among which SFN could effectively inhibit various sorts of HDAC, which was commonly accompanied with decreased DNMTs, to induce autophagy, apoptosis and cell cycle alterations in different cancers. SFN has been shown to limit the total activation and global protein level of HDAC1-4, as well as directly enhance the nuclear translocation of NRF2 and, as a result, upregulate cellular defense enzymes HO-1 and NQO1, acting as an anti-cancer agent against neoplastic transformation of mouse skin JB6 P+ cells (Su et al., 2014). Additionally, SFN also indirectly upregulated NRF2 expression by enhancing binding between NFE2L2 promoter and active chromatin marker acetylated histone 3 (Ac-H3), while the protein level of Ac-H3 could be increased by prominently attenuated HDAC1, 4, 5, and 7 or impaired formation of the transcriptional regulator complex partly consisted of DNMT and HDAC after SFN treatment in prostate cancer (Zhang et al., 2013) and breast cancer (Sharma et al., 2005; Saxena and Sharma, 2010; Meeran et al., 2012) respectively, restoring the cellular antioxidant and detoxification effects.

Besides, disparate bioactive dietary supplements regulate NRF2/KEAP1 pathway *via* targeting histone acetylation. CRA was observed to prevent cellular damage and maintain tissue homeostasis *via* restricting class I and II HDAC thus activating NRF2 in the prostate cancer (Chen et al., 2012; Yang et al., 2018a). Furthermore, demethylated CpGs in NFE2L2 promoter region on account of decreased HDACs synergistically accompanied with attenuated DNMTs after treatment with DIM in prostate cancer (Lewinska et al., 2017) or Delphinidin in the mouse skin JB6 P+ cells (Kuo et al., 2019) was observed, contributing to enhanced expression of NRF2 and its downstream target gene such as HMOX1, NQO1 and SOD1, ultimately inducing different anticancer effects such as the upregulated detoxification and excretion of chemicals (Nioi and Hayes, 2004) or attenuated cell transformation (Kuo et al., 2019). In addition, a clinic trial conducted by Barbara Ann Karmanos Cancer Institute (NCT00888654) confirmed that the use of DIM may slow the growth of tumor in 41 patients with stage I or stage II prostate cancer undergoing radical prostatectomy. Other than these, diverse cancer chemopreventive agents like pelargonidin (Li et al., 2019), taxifolin (TAX) (Kuang et al., 2017) and Ursolic acid (UA) (Kim et al., 2016) have been proven to epigenetically diminish HDACs and reactivate NRF2/KEAP1 pathway to exert anticancer effects in the mouse skin JB6 P+ cells, while CRA in NSCLC (Jin et al., 2021) and LUT in colorectal cancer (Zuo et al., 2018) have been observed to target the same signal pathway, subsequently inducing downstream target genes with respect to cell cycle arrest, autophagy and apoptosis in cancer cells.

Apart from histone acetylation towards NFE2L2, Alam et al. (2017) have elaborated that glucocorticoids (GC) could directly inhibit histone acetylation at ARE and decrease NRF2 transcriptional activation through glucocorticoid receptor (GR) signaling, resulting in impaired NRF2-mediated antioxidant response due to the side effects of GC in hepatocellular carcinoma.

### 4.3 Targeting ncRNA

Besides DNA methylation and histone acetylation, ncRNAs widely involve in posttranslational gene modification, regulating the pathophysiological processes of cells (Bandres et al., 2009). MiRNA, the most common and effective ncRNA, has been substantiated to epigenetically regulate NRF2/KEAP1 signaling, considered as a hallmark of cancer (Duthie, 2011; Sandoval and Esteller, 2012). Some investigators have implicated that various dietary bioactive compounds could potently control the aberrant expression of miRNAs, contributing to the activation or silence of downstream genes. Here, we reviewed phytochemical-based cancer treatments targeting miRNAs in NRF2/KEAP1 signaling.

As one of the most powerful phytochemicals, the role of ITC as a potent miRNAs regulator resulting in preventing tumor incidence has received intense attention. In prostate cancer model, PEITC remarkably elevated miR-200c (Gerhauser, 2013) while diminished both pri-miR-141 and mature miR-141 expression (Xiao et al., 2012), reducing tumor metastasis by nearly 50%, inhibiting ROS production as well as inducing apoptotic cell death (Chiao et al., 2004). In addition, it has been confirmed that miR-200a-3p/141-3p directly combined to 3′-UTR of KEAP1, thus profoundly dysregulating NRF2/KEAP1 pathway in renal tumorigenesis and ovarian cancer cells, which could be blocked by PEITC significantly to reduce the oxidative stress response (Mateescu et al., 2011; Lan, 2012; Jiang et al., 2013; Cortez et al., 2014). A clinic trail conducted by Portland VA Medical Center (NCT01265953) that enrolled 98 patients suffering from prostate cancer has not only proven the protective role of ITC in inhibiting cancer development, but also identified the altered gene expression caused by epigenetic modifications.

In addition to ITC, other chemopreventive compounds exerted anti-tumorous effects *via* regulating miRNA in various cancers. In breast cancer model, miR-93 could decrease NRF2 expression at mRNA and protein levels (Singh et al., 2013) to impair its downstream genes such as NQO1 and SOD3 (Singh and Bhat, 2012; Singh et al., 2012), thus playing a crucial role in regulating apoptosis and oxidative DNA damage in cancer cells. Singh et al. (2014) has elaborated that in combination with 17β-estradiol (E2), Resveratrol treatment inhibited expression of NRF2 targeting miR-93 and upregulated NRF2 promoter methylation, substantially attenuating cellular proliferative changes and tumor development. Similarly, as a dietary supplement expected to prevent oxidative stress-mediated chronic diseases, Vitamin C prevented E2-mediated miR-93 overexpression to upregulate NRF2 and its downstream NQO1, consequently exerting defensive effect against oxidative DNA damage and E2-induced mammary tumorigenesis in mouse model (Mense et al., 2009; Singh et al., 2012; Singh et al., 2013). Intriguingly, Wang et al. (2016b) also found the antineoplastic effect of Vitamin C *via* regulating miR-153 in E2-treated human mammary cell lines, but the specific mechanism needed further exploration.

Other dietary phytochemicals also made a difference in regulating miRNAs and subsequently targeting NRF2/KEAP1 signaling. In hepatocellular carcinoma, accumulating evidence indicated that Apigenin negatively regulated the protein level of NRF2 through inducing miR-101, which could directly target the 3′-UTR of NRF2 (Gao et al., 2017) to ultimately suppress cancer apoptosis and oxidative damage (Liu et al., 2016). Coincidentally, Zhao et al. (2018) has proven that Polydatin (3,4′,5-trihydroxy-stilbene-3-β-D-glucoside) upregulated miR-200a to target the 3′-UTR of KEAP1, activating NRF2 and its target genes, accordingly restraining liver inflammation and lipid deposition. Those findings provided us that various dietary phytochemicals could influence miRNA expression level to regulate NRF2/KEAP1 pathway, acting as essential parts in anti-oxidative and anti-tumorous microenvironment.

## 5 Conclusion

NRF2/KEAP1 signaling plays an important role in modifying oxidative stress, which is subjected to various regulations at transcriptional, translational and post-translational levels. A growing body of recent evidence shows that NRF2 and KEAP1 expression can be regulated by CpGs methylation/demethylation, histones acetylation/deacetylation and ncRNAs. In addition, the association between NRF2/KEAP1 signaling and cancer aggressiveness is certain. Thus, targeting the epigenetic modifications of NRF2/KEAP1 signaling is suggested as a feasible and promising therapeutic approach for cancer in three aspects. First, genetic mutations are permanent while epigenetic abnormalities are reversible, which offers a potential opportunity to revert it with agents or drugs. Second, there are many FDA-approved epigenetic therapies and ongoing investigation about second generation of novel epigenetic therapies for cancer treatment (Grønbaek et al., 2007; Dhanak and Jackson, 2014). Third, the consumption of dietary phytochemicals is proved to prevent cancer and have anticancer effects through altering epigenetic modifications.

However, the dual roles of NRF2/KEAP1 signaling in cancer must be taken into consideration. During early stages of tumorigenesis, oxidative stress has been shown to increase the frequency of DNA mutation, which in turn, contributes to tumor initiation. Therefore, enhancement of NRF2 activity is desirable to combat oxidative stress and prevent malignant transformation in premalignant states. While once a tumor is formed, the same defense system can also be utilized by fully malignant cancer cells to create a reductive microenvironment which is beneficial to rapid proliferation and therapy resistance of tumor (Sporn and Liby, 2012). Apart from different cancer stages, it is also critical to consider the temporal nature of NRF2/KEAP1 signaling. Transient NRF2 induction in an acute xenobiotic exposure can initiate vital stress response pathways and provide cytoprotection for chemopreventive purposes. Dissimilarly, chronic toxicant exposure, mutations and epigenetic modifications have all been demonstrated to activate NRF2 in a prolonged manner, resulting in tumor progression (Dodson and Zhang, 2017). Therefore, a deeper understanding of contextual and temporal control of NRF2 will allow the optimal development of new drugs targeting epigenetic modifications of NRF2/KEAP1 signaling.
